# Prevalence and risk factors of perinatal depression among women in rural Bihar: A community-based cross-sectional study

**DOI:** 10.1016/j.ajp.2021.102552

**Published:** 2021-02

**Authors:** Vijaya Raghavan, Homam A. Khan, Uttara Seshu, Surya Prakash Rai, Jothilakshmai Durairaj, G. Aarthi, C. Sangeetha, Sujit John, R. Thara

**Affiliations:** a Schizophrenia Research Foundation, R/7A, North Main Road, Anna Nagar West Extension, Chennai, 600101, Tamil Nadu, India; b Innovators In Health (India) Nagar Panchayat, Ward 02, Thana: Dalsinghsarai, Distt: Samastipur, Bihar, 848114, India

**Keywords:** Perinatal depression, Prevalence, Risk factors, Physical health, Abortion, Poverty, Community survey, Rural

## Abstract

**Background:**

Perinatal depression (PND) is one of the most common mental disorders occurring during the perinatal period among women. Few studies examined prevalence and risk factors of PND from rural settings in India. This study aimed to estimate the prevalence of perinatal depression and identify social risk factors for it among women from rural Bihar.

**Materials and methods:**

A cross sectional study was conducted in a community setting in rural areas of Bihar. All perinatal women were screened through a door to door survey and recruited after obtaining informed consent. A semi-structured proforma was used to collect sociodemographic characteristics and family related variables. Edinburgh postnatal depression scale (EPDS) was used to screen for perinatal depression.

**Results:**

A total of 564 perinatal women were recruited into the study. The estimated prevalence of PND was 23.9 % (95 % CI: 20.6,27.6). Multivariate analysis showed perinatal depression was associated with physical illness in the mother, previous history of abortion, poor financial status and ill-treatment by in-laws.

**Conclusion:**

Prevalence of perinatal depression among women is high in rural settings of North India. A multitude of factors ranging from physical, obstetric, economic and family related confer a high risk for PND. Comprehensive interventions are needed to address these risk factors of perinatal depression.

## Highlights

• Estimated prevalence of perinatal depression in rural Bihar, India was 23.9%.
• Perinatal depression is associated with mother’s physical illness and previous abortions.
• Poor financial status and ill-treatment by in-laws predicted perinatal depression.

## 1 Introduction

Pregnancy and postnatal period are two of the most vulnerable time periods in a woman’s life (Briscoe et al., 2016; Johnston-Ataata et al., 2018). During the antenatal and postnatal period, physiological (Soma-Pillay et al., 2016) and psychological (Leifer, 1977) changes occur, leading to an increased risk of both physical and mental health issues among women (Howard et al., 2014; O’Hara and Wisner, 2014). These health issues have an impact not only on the maternal health and pregnancy outcomes but also on the short- and long-term developmental trajectories of the child (Burger et al., 2020; Hoffman et al., 2017; Stein et al., 2014).

Perinatal depression (PND), defined as the depression in women during pregnancy or within 12 months of delivery, is probably the most common mental health disorder in women (Muzik and Borovska, 2010). It is a significant mental and public health problem (Shrivastava et al., 2015). PND is associated with many adverse sequelae for the woman, her family and children. These could be poor maternal-fetal attachment, adverse neonatal outcomes (low birth weight, preterm birth, small for gestational age), early childhood developmental delays, or relationship strain (Alhusen et al., 2013; Grigoriadis et al., 2013; Leis et al., 2014).

Studies show the prevalence of postnatal depression ranges from 15 to 35% with an average of around 19 % in low- and middle- income countries (Gelaye et al., 2016). Indian studies have reported the prevalence of antenatal depression to range from 9.18%–65.0% (Arora and Aeri, 2019). In other parts of the world, it is around 25 % (Biratu and Haile, 2015; Weobong et al., 2014; Zeng et al., 2015). These rates may however vary with different geographical and socioeconomic l factors.

The major risk factors for perinatal depression among women include past history of depression, presence of anxiety, marital difficulties or lack of a partner, lack of social support and recent major life events (Ghaedrahmati et al., 2017; Lancaster et al., 2010). Poverty, substance abuse, previous abortion, unplanned pregnancy, family violence, ambivalence towards the pregnancy and history of abuse may also contribute (Smorti et al., 2019).

The studies from India that have examined the prevalence and risk factors in prenatal and postnatal depression have been largely hospital-based (Dash, 2020; Johnson et al., 2018; Modi et al., 2018). Community based studies have been very few, much less in rural parts of the northern part of India. Hence, the aim of the study was to estimate the prevalence of perinatal depression and to identify the risk factors associated with perinatal depression among women residing in rural Bihar, India.

## 2 Materials and methods

### 2.1 Site

We conducted the study in Dalsinghsarai Taluk, Samastipur District, Bihar, India. Bihar is one of the underdeveloped states in India with a high percentage of illiteracy and poor economic development. The total population of Bihar is 10,38,05,000 and the population of the Dalsinghsarai is 208,818 (Chandramouli and General, 2011). Bihar spends just 2% of state GDP on health as compared to other states that spend about 6–8 %. Bihar’s maternal mortality rate of 208 per 100 000 live births is much higher than the national average of 167 per 100 000 live births. The infant mortality rate is also high. Thus, Bihar has poor health budgetary allocations, and poor health outcomes and indicators.

In addition, mental health is low priority for the State and does not feature in the annual health budget, except for the revenue allocation under centrally sponsored scheme NMHP-DMHP that is operational in only 11 out of 38 districts. The NHRC provided a summary of specialized human resources for mental healthcare in Bihar, which was very inadequate. The number of psychiatrists trained every year is also less than 10.

### 2.2 Study participants

All perinatal women (those currently pregnant or within a year after delivery/abortion) in the catchment area were identified using a door to door survey method. The survey was done by the community level health workers who were trained in the concepts of mental health and mental disorders and perinatal depression along with administering screening tool. Those who gave informed consent were included for the study.

### 2.3 Assessment tools

Sociodemographic and family related variables: We developed a semi-structured proforma to collect information on the sociodemographic profile, physical, obstetric, marital/family related and economic variables.

Edinburgh postnatal depression scale (Cox et al., 1987): Edinburgh postnatal depression scale (EPDS) is a 10-item self-report scale to screen for postnatal depression. The scoring is based on a 1-week recall of the symptoms. Though primarily developed for the detection of postnatal depression, EPDS can be used for the screening of perinatal depression also (Milgrom and Gemmill, 2014). Each item is rated from 0 to 3, yielding a total score of 0–30. We used a Hindi translation of EPDS in the study. A score of 10 or above in EPDS indicates possible depression, based on the cut-off scores provided by the scale and a previous study on perinatal depression from India (Rathod et al., 2018).

### 2.4 Administration of the tools

All the tools were administered by the trained community level workers (CLWs). Before the start of the data collection, the CLWs were trained by the psychiatrist and psychiatric social worker. During the training, CLWs were educated on mental health and specific mental health disorders occurring during pregnancy and post-partum period. They were trained to identify the early symptoms of major mental health disorders among perinatal women including depression and psychosis, initiate proper referral and follow-up. CLWs were also trained to administer the EPDS the study participants. The interrater reliability among the CLWs for the tools administered by them was satisfactory (Cohen's kappa coefficient, κ = 0.7).

### 2.5 Ethical considerations

The study protocol was approved by the institutional review board and ethics committee of Schizophrenia Research Foundation, Chennai, Tamil Nadu, India. The study was initiated after the ethical approval. Informed written consent was obtained from the voluntary study participants in the local language (Hindi) before recruiting them into the study. All the study participants were provided with the information on and the resources available for the treatment of antenatal and postnatal depression. Women screened positive in the EPDS (score of 10 or above) were provided expert care under a trained psychiatrist and appropriate management including pharmacological and non-pharmacological treatments, as needed. Anonymity and confidentiality were maintained all through the process.

### 2.6 Statistical analysis

The prevalence of perinatal depression was presented with 95 % CI. Continuous and categorical socio-demographic profiles were summarized as mean with SD and frequencies with percentages were provided. The categorical variables were associated with the outcome (Perinatal depression and No perinatal depression) using the chi-square test. All continuous variables were compared across the groups using an independent *t*-test or Mann Whitney *U* test as appropriate. Logistic regression analysis was done adjusting for confounders and risk factors. p < 0.05 was considered statistically significant. Data were analyzed using SPSS software version 16.0.

## 3 Results

The summary measures of all socio-demographic variables are provided in Table 1. The mean (SD) age of the study participants is 23.4 (3.7). Table 2 presents the prevalence of perinatal depression among the study participants and it was found to be 23.9 % (95 % CI: 20.6,27.6).

**Table 1:** Sociodemographic profile of the study participants (N = 564).

Variable	Mean (SD); N (%)
Age (in years)	23.4 ± 3.7
Education (in years)	6.2 ± 4.8
Monthly income of the household	
< 5000	245 (43.3)
5000 – 9000	208 (36.7)
10,000 -15,000	100 (17.7)
>15,000	13 (2.3)
Number of children	
0	99 (17.6)
1-3	405 (71.8)
>3	60 (10.6)
Mean	1.9 ± 1.5
Age during first marriage	16.7 ± 2.7
Age during first pregnancy	18.8 ± 3.4
Abortion	
No	534 (94.7)
Yes	30 (5.3)
Still birth	
No	547 (97)
Yes	17 (3)

**Table 2:** Prevalence of perinatal depression among the study participants (N = 564).

EPDS	N (%)	95 % CI
No	429 (76.1)	72.8–79.8
Yes	135 (23.9)	20.6–27.6

The results of the socio-demographic, physical health, family and economic factors are presented in Table 3. Previous abortions, (16.3 Vs 1.9 %; p < 0.001). ill-treatment by in-law's family, physical abuse, lack of support, husband working away from home in major cities, living alone, poor financial status and presence of physical illness were all highly associated with perinatal depression.

**Table 3:** Univariate analysis of the factors associated with perinatal depression among study participants (N = 564).

Variable	Perinatal depression (N = 135) N (%)	No perinatal depression (N = 429) N (%)	p-value
Age	23.8 ± 3.8	23.3 ± 3.7	0.145
Age during first marriage	16.6 ± 2.9	16.9 ± 2.6	0.020
Age during first pregnancy	18.4 ± 3.5	18.9 ± 3.4	0.162
Previous abortion			
No	113 (83.7)	421 (98.1)	<0.001
Yes	22 (16.3)	8 (1.9)	
Still birth			
No	128 (84.8)	419 (97.7)	0.143
Yes	7 (5,2)	10 (2.3)	
Ill treatment by in-law family			
No	85(63)	411 (95.8)	<0.001
Yes	50 (37)	18 (4.2)	
Physical abuse			
No	94 (69.9)	417 (97.2)	<0.001
Yes	41 (30.4)	12 (2.8)	
Lack of support			
No	83 (61.5)	402 (93.7)	<0.001
Yes	52 (38.5)	27 (6.3)	
Husband working away			
No	97 (71.9)	356 (83.4)	0.004
Yes	38 (28.1)	71 (16.6)	
Living alone			
No	105 (77.8)	408 (95.1)	<0.001
Yes	30 (22.2)	21 (4.9)	
Weak financial status/debts			
No	78 (57.8)	385 (89.7)	<0.001
Yes	57 (42.2)	44 (10.3)	
Physical illness in mother			
No	115 (85.2)	423 (98.6)	<0.001
Yes	20 (14.8)	6 (1.4)	
First pregnancy			
No	112 (83)	359 (83.7)	0.878
Yes	23 (17)	70 (16.3)	

The adjusted analysis was provided for the variables which were significant in univariate analysis in Table 4. The odds of perinatal depression were 4.62 times higher among the women who experienced the Ill-treatment by in-law's family as compared to those who did not (p < 0.001). 2.47 times higher among the women with weak financial status (p = 0.003) and 7.14 times higher when physical illness was present (p < 0.001). It was also 3.76 times higher among those who had previous abortions (p = 0.009).

**Table 4:** Multivariate analysis using logistic regression to identify the risk factors for perinatal depression among study participants (N = 564).

Variable	OR (95 % CI)	P value
Ill treatment by in-law family		
No	1.00	
Yes	4.62 (2.03, 10.54)	<0.001
Weak financial status/debts		
No	1.00	
Yes	2.47 1.36,4.52)	0.003
Physical illness in mother		
No	1.00	
Yes	7.14(2.56,19.91)	<0.001
Previous abortion		
No	1.00	
Yes	3.76(1.31,10.82)	0.009

## 4 Discussion

The aim of our study was to estimate the prevalence of perinatal depression and the risk factors associated with PND among women in rural Bihar, North India.

The prevalence of PND was 23.9 %. Previous studies from India has shown wide variations in the prevalence of perinatal depression. Rathod et al., in 2018 found the prevalence of PND to be 18.5 % in the clinical facility sample while it was 8.8 % from the community sample. Shivaji and Gururaj in 2015 reported a rate of 31.4 % in a sample from south India. (Shivalli and Gururaj, 2015). A meta-analysis from India found a rate of 22 % (Upadhyay et al., 2017). This seems to be more comparable with our figure of 23.9 %. Another meta-analysis from low- and middle- income countries, found a prevalence of around 19.6 % for PND (Gelaye et al., 2016).

Various factors such as location of the study and tools used could account for the wide variations in the reported prevalence of perinatal depression in India. A slightly higher rate in our study This difference could be due to the fact that the women being from a rural disadvantaged community had to contend with multiple social risk factors contributing for depression. Other studies from the disadvantaged communities have also shown higher prevalence of perinatal depression when compared to the national average (Jaju et al., 2015; Shivalli and Gururaj, 2015).

We observed that physical illness in the mother increased risk of perinatal depression eight-fold. This has been observed by many others as well (Brown et al., 2018, 2019). Poverty, poor social support, harassment by in laws and poor access to health services could all play havoc with a woman’s health. This underscores the need to adopt a comprehensive approach to health care.

Previous abortion was significantly associated with perinatal depression. Many previous studies from India (Ajinkya et al., 2013) and other parts of the world have shown similar findings (Asaye et al., 2020; Ludermir et al., 2011). Past pregnancy loss is not only associated with PND, but also with other psychiatric disorders such as anxiety and post-traumatic stress disorder (Giannandrea et al., 2013). Abortions can cause grief like reactions, worsening of physical health which in turn can increase the risk of perinatal depression. It could well be noted that antenatal depression and anxiety can also lead to abortion (Biaggi et al., 2016).

Weak financial status with or without debt resulting in poverty and chronic stress is a powerful predictor of PND as shown by Patel and others (Patel et al., 2003, 2002; Shivalli and Gururaj, 2015). Ill treatment by in laws has been identified by others also as a predictor of PND (Arora and Aeri, 2019; Jaju et al., 2015; Shidhaye et al., 2017). Physical abuse and lack of social support from others did not emerge to be significant in our study. However, this association has been widely reported by many others (Kitamura et al., 2004). Intimate partner violence (Beydoun et al., 2010) and poor relationship with partners have been shown to predict PND.

Major strengths of this study are that it was a community-based study recruiting all the perinatal women in the study site using door-to-door surveys; We used a standardized instrument to screen for perinatal depression. Our sample size is quite large, and we imparted extensive training to the CLWs and assured their fidelity in the administration of EPDS.

Major limitations of the study are: Standard tools to collect family and economic variables could not be used. This information was collected as subjective reports. We had to minimize the interview time and the number of tools used due to lack of time and feasibility of conducting confidential interviews in these settings; The physical health parameters such as hemoglobin, nutritional status, blood pressure and gestational diabetes were not measured in the study due to the lack of facilities available to assess these measures. Being a cross-sectional design, we could arrive only at meaningful associations but not causal directions of associations.

Longitudinal studies are needed to estimate the incidence and examine risk and protective factors that contribute to perinatal depression. Based on these results, interventional studies targeting important, locally relevant risk and protective factors need to test for the prevention of perinatal depression leading to better maternal and child outcomes in the short- and long-term.

## Source of funding

This work was support by Grand Challenges Exploration (GCE-USA) Round 21, 10.13039/100000865Bill and Melinda Gates Foundation.

## Declaration of Competing Interest

Nil.
